# Obturator Compartment Syndrome Secondary to Pelvic Hematoma After Robot-Assisted Laparoscopic Radical Prostatectomy

**DOI:** 10.1089/cren.2016.0075

**Published:** 2016-08-01

**Authors:** Jun H. Song, Joshua R. Kaplan, Daniel Abbott, Eric Gewirtz, Ellen Hauck, Daniel D. Eun

**Affiliations:** ^1^ Temple University School of Medicine, Philadelphia, Pennsylvania.; ^2^ Department of Urology, Temple University School of Medicine, Philadelphia, Pennsylvania.; ^3^ Department of Anesthesiology, Temple University School of Medicine, Philadelphia, Pennsylvania.

**Keywords:** robotics, prostate cancer, RALP

## Abstract

Obturator nerve injury is a known injury after robot-assisted laparoscopic radical prostatectomy (RALP) and patients often present with motor and sensory deficits in the immediate postoperative period. We describe a 65-year-old male who presented with motor deficits, indicative of obturator neurapraxia after RALP upon waking from anesthesia. Work-up revealed an expansile hematoma possibly compressing the obturator nerve. After evacuation of the hematoma, the patient had immediate improvement of his neurologic deficits. Our patient's clinical vignette illustrates the importance of considering postsurgical hematoma in the differential diagnosis when patients present with signs and symptoms of obturator neurapraxia after RALP.

## Introduction

Obturator nerve injury is an inherent risk during robot-assisted laparoscopic radical prostatectomy (RALP). This injury is typically in the form of stretch, burn, or transection of the nerve. An injury to the obturator nerve clinically presents with diminished sensation in the medial thigh, pain in the groin area and medial thigh, and weakness in ipsilateral leg adduction that is immediately apparent in the recovery room.^1^ We present a case of postoperative obturator neurapraxia secondary to a left pelvic gutter hematoma and not from direct nerve trauma after RALP with bilateral pelvic lymphadenectomy.

## Case Presentation

A 65-year-old male was found to have clinical stage T1c prostate cancer after demonstrating multicore Gleason 3 + 4 on prostate biopsy with a PSA of 5.5 ng/dL. The patient underwent an uneventful RALP with bilateral pelvic lymph node dissection with an estimated blood loss of 100 mL. Bilateral dissection of the obturator and external iliac nodes went smoothly and bilateral nerves were noted to be intact at the end of surgery. Furthermore, the patient was hemodynamically stable throughout the case and at the conclusion of surgery. There were no reported patient sliding issues and positioning was Trendelenburg and supine, without lithotomy or stirrups, using the da Vinci Xi console docked from the side. In the post-anesthesia care unit (PACU), the patient became progressively hypotensive (BP 88/46) and complained of groin and thigh discomfort. In addition, anesthesiology noted that the patient demonstrated left leg weakness and an inability to lift and adduct his left leg. Since there was no known obturator nerve injury, it was initially postulated that the patient may have had a femoral nerve stretch injury possibly related to positioning or unrecognized sliding on the table. Right lower extremity examination was completely normal and neural examination was intact. As the surgical team was notified of the neurologic findings, the video file was immediately reviewed that confirmed that there was no trauma, clipping, stretching, or thermal injury to the obturator nerves. In addition, although his preoperative hemoglobin and hematocrit were within normal limits (15.3 g/dL and 46.3, respectively), his postoperative hemoglobin and hematocrit dropped below the normal range (9.8 g/dL, and 28.4, respectively). The patient was transfused with 2 units of packed red blood cells on postoperative day (POD) 1. The patient continued to complain of thigh and groin pain, and the neurologic deficits did not improve. He also developed a left lower quadrant flank hematoma. Because of this clinical course, a CT scan of the abdomen and pelvis was performed, which showed an 8.7 × 3.3 × 7.5 cm hyperdense collection in the left hemipelvis. This finding was consistent with a large postoperative hematoma along the left pelvic gutter, involving the obturator fossa. Neurology was consulted on POD 1 and agreed with the obturator nerve deficit findings. The patient was taken back to the operating room (OR) on POD 2 for a robot-assisted exploration and hematoma evacuation. A percutaneous drainage was not indicated for this patient because of a suspicion that he may be actively bleeding and a drain would likely not effectively drain the hematoma. Approximately 300 to 400 mL of organizing clot was evacuated from the obturator fossa. In addition, the obturator artery was found to be the likely site of the recent bleed and was clipped. In the PACU, the patient had an immediate improvement in his neurologic symptoms, regaining ∼75% of normal motor strength. His hemoglobin remained stable and was discharged home on POD 7 in stable condition. He was seen 4 weeks postoperatively and reported ∼95% of normal motor strength.

## Discussion

Obturator neuropathies are serious complications after RALP. These complications are usually a direct result of physical nerve damage intraoperatively, which will present clinically with motor and sensory deficits to the lower limb.^1^ Although uncommon, most neuropathies that develop as a result of intraoperative injury resolve within a few weeks unless complete transection or ligation occurs.^2^ Stretch and partial nerve injuries are often treated conservatively through pharmacologic management, physical therapy, and rest. In this case, however, given the acute neurologic findings in the absence of direct nerve injury, the surgical team decided to take the patient back into the OR to rule out hematoma-related compartment syndrome.

Our patient first developed signs of obturator neurapraxia in the postanesthesia area after undergoing RALP with pelvic lymphadenectomy. Koç et al. attribute most neuropathies present in the immediate postoperative period to intraoperative complications.^2^ However, operative video review revealed no evidence of nerve injury. A possible explanation of the patient's symptoms could have been caused by positioning during surgery as this can also cause transient neuropathies postoperatively. More specifically, lower extremity neuropathy in patients placed in supine position has been discussed in the literature.^3^ Therefore, since the patient had been placed in supine position for the procedure, a transient obturator neurapraxia caused by positional compression or stretch injury of the obturator nerve was high on the differential diagnosis, but ultimately proved not to be the case.

**FIG. 1.**
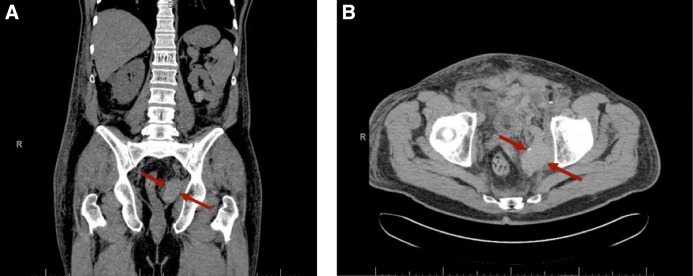
Postoperative CT scans. **(A)** Coronal section. **(B)** Axial section. *Red arrows* indicate expansile hematoma in the left paracolic gutter.

The patient's postoperative drop in blood pressure, hemoglobin, and hematocrit was also a cause for concern. Despite transfusion of 2 units of packed red blood cells, the patient's hemoglobin and hematocrit remained low. A CT scan (Fig. 1) showed a hyperdense collection in the left pelvis, indicative of a left deep pelvic hematoma. To our knowledge, although femoral neuropathies secondary to hematoma compression have been described, obturator neurapraxia has not been discussed in the literature.^4^ Patients with femoral neurapraxia present similarly with motor and sensory deficits to the lower limbs. After consideration of the clinical findings, our patient was found to have an obturator neurapraxia secondary to a left deep pelvic hematoma, which was confirmed upon robot-assisted laparoscopic re-exploration on POD 2. After the pelvic hematoma was evacuated, the patient regained his left lower motor and sensory functions.

Obturator nerve compartment syndrome is likely a rare complication, but should be included on the differential diagnosis in patients who present clinically with neurologic deficits of the lower limb after an uneventful radical prostatectomy. CT work-up could be done for patients with these symptoms in the context of a hematocrit drop to assess the possibility of a hematoma compressing on the obturator nerve. Hematoma evacuation should be considered if the patient's symptoms are persistent or worsening in the setting of a collection in the area of the obturator foramen. Furthermore, if any bleeding complications during or after RALP occur, surgeons should carefully monitor the patient's motor and sensory functions to the lower limb in case of any possible nerve impingement by a pelvic hematoma. A correct and early diagnosis of neurapraxia secondary to a hematoma may be critical in regaining neurologic function in patients who present with lower limb neurologic deficits after RALP.

## Conclusion

Although rare, neurologic complications after RALP could be a direct result of postoperative bleeding. To our knowledge, we describe the first obturator nerve compartment syndrome by an expansile pelvic gutter hematoma. Postoperative patients with a pelvic hematoma presenting with obturator nerve deficits in the absence of direct nerve injury should be taken back to the OR for exploration and clot evacuation.

## Abbreviations Used

CT: computed tomography
OR: operating room
POD: postoperative day
PSA: prostate specific antigen
RALP: robot-assisted laparoscopic radical prostatectomy
